# Extraction of Dairy Phospholipids Using Switchable Solvents: A Feasibility Study

**DOI:** 10.3390/foods8070265

**Published:** 2019-07-18

**Authors:** Shouyun Cheng, Kaavya Rathnakumar, Sergio I. Martínez-Monteagudo

**Affiliations:** Dairy and Food Science Department, South Dakota State University, Alfred Dairy Science Hall, Brookings, SD 57007, USA

**Keywords:** phospholipids, N,N-dimethylcyclohexylamine, switchable hydrophilicity solvent, dairy byproducts

## Abstract

A tertiary amine (N,N-dimethylcyclohexylamine, CyNMe2) was used as a switchable hydrophilicity solvent (SHS) for extracting phospholipids (PLs) from raw cream (RC), buttermilk (BM), concentrated buttermilk (CBM), and beta-serum (BS). The SHS extractions were performed with varying solvent–sample weight ratio at room temperature. The extracted PLs using CyNMe2 were recovered by bubbling CO_2_ at atmospheric pressure, switching the CyNMe2 into its respective salt. For comparison, the PLs were also extracted using Folch (FE) and Mojonnier (ME) extraction. The extraction efficiency of SHS varied from 0.33% to 99%, depending on the type of byproduct. The SHS extracted up to 99% of the PLs directly from BM, while only 11.37% ± 0.57% and 2.66% ± 0.56% of the PLs were extracted with FE and ME, respectively. These results demonstrate the applicability of SHS for the extraction of PLs from dairy byproducts.

## 1. Introduction

Phospholipids (PLs) are found as lipid bilayers in all plant and animal cell membranes [1]. The term PLs refers to a class of complex polar lipids containing a phosphate group and two fatty acids esterified to a glycerol backbone. The phosphate group is linked to a polar group such as choline, ethanolamine, or serine [2]. PLs derived from milk are primarily rich in sphingomyelin (SM) and phosphatidylserine (PS), two of the most highly bioactive PLs [3]. Health benefits associated with the consumption of PLs include reduced incidence of cardiovascular diseases, cholesterol adsorption, reduced gastrointestinal infections, and improved immune function [1,4].

Dairy PLs are embedded with epithelial cell plasma membranes, forming a complex structure known as the milk fat globule membrane (MFGM). Such arrangements allow the milk fat to be emulsified and dispersed within the milk [5]. In raw milk, PLs account for about 0.5%–1% of the total milk fat, and about 60%–70% of the PLs are located in the MFGM, depending on the variety, season, and lactation stage [2]. On the other hand, the content of PLs in dairy foods is strongly influenced by the manufacturing steps [6]. An investigation measuring the concentration of PLs in 31 dairy foods showed values of PLs ranging from 0.1% to 25% of the total milk fat [7]. 

Dairy byproducts represent a natural source of PLs with great potential for isolation and further commercialization. The isolation of PLs from byproduct streams involves various steps (i.e., concentration, extraction, solvent separation, lipid recovery, and fractionation) within the entire process, which results in low overall efficiency and is therefore economically unviable. Instead, concentrates of PLs obtained from dairy byproducts such as buttermilk have been a research priority in the past few years [7,8,9,10]. As previously highlighted, the applicability of PL concentrates is limited in comparison with the industrial application of PLs derived from lecithin [11]. Thus, it is desirable to develop technology for producing isolated PL fractions derived from dairy byproducts. 

Recently, a new class of solvents—namely, switchable hydrophilicity solvents (SHS)—have been developed to facilitate the extraction and subsequent separation of lipidic materials [12]. These types of solvents are made from primary, secondary, or tertiary amines, and they are capable of extracting lipids from wet materials. SHSs abruptly and reversibly switch between a hydrophobic form (poorly miscible with water) and a hydrophilic form (miscible with water). The polarity switch of these solvents is triggered by simply bubbling or removing CO_2_ [13]. The underlying principle behind the change in polarity is due to the formation of an ammonium carbonate salt in the presence of CO_2_, while upon removing the CO_2_ with nitrogen, the carbonate salt returns to its original amine form. Earlier, an investigation employed N,N,N’-tributylpentanamidine as an SHS for the extraction of soybean oil [13]. Similarly, other authors extracted lipids from freeze-dried microalgae using a tertiary amine (N,N-dimethylcyclohexylamine, CyNMe2) as an SHS [14]. More importantly, the SHS was removed from the extract without distillation. In summary, the existing literature on the extraction of lipids via SHSs reveals that CyNMe2—a commercially available amine—has tunable hydrophilicity, meaning that it can be switched from a hydrophobic form into a hydrophilic form by adding CO_2_. The present research aimed to evaluate the feasibility of the extraction and separation of PLs from different dairy matrices using CyNMe2 as a switchable hydrophilicity solvent.

## 2. Materials and Methods 

### 2.1. Dairy Byproducts

The tested dairy matrices were raw cream (RC), buttermilk (BM), concentrated buttermilk (CBM), and beta-serum (BS). The RC was obtained from the Davis Dairy Plant at South Dakota State University (Brookings, SD, USA). A portion of the cream was churned at 4 °C to obtain BM using a laboratory-scale churner (TM 31 Thermomix, Vorwerk LLC, Thousand Oaks, CA, USA). The CBM was obtained by freeze-drying BM using a benchtop freeze drier (Model 117, Labconco Corporation, Kansas City, MO, USA). The BS was obtained from a local plant (Valley Queen, Milbank, SD, USA). N,N-dimethylcyclohexylamine (99%, Sigma Aldrich, St. Louis, MO, USA), phospholipid mixture for HPLC (Soybean, P3817-1VL, Sigma Aldrich ), methanol (99.9%, Sigma Aldrich), chloroform (99.9%, Sigma Aldrich), hexane (99.9%, Sigma Aldrich), HPLC-grade water (Sigma Aldrich), histological grade ethanol (Sigma Aldrich), petroleum ether (95%, Fisher Scientific), ethyl ester (99%, Fisher Scientific), phosphomolybdic acid hydrate (99%, Alfa Aesar), TLC silica gel plate (TLC silica gel 60 F254, EMD Millipore, Burlington, MA, USA), and activated silica gel (Silica gel 60 G, EMD Millipore) were purchased from commercial suppliers.

### 2.2. Compositional Analysis

A sample of each matrix was tested for total solids, protein, fat, lactose, and pH. The total solids were determined gravimetrically. The protein content on a total nitrogen basis was determined by the Kjeldahl method. The fat content was measured gravimetrically according to the method of Mojonnier fat extraction. The concentration of lactose was determined using HPLC following the methodology reported elsewhere [15]. The pH was measured in 10 mL of the sample using an Orion Versa Star Pro (Thermo Fisher Scientific, Waltham, MA, USA).

### 2.3. Conventional Extraction

The extraction of total lipids was performed using Folch (FE) and Mojonnier (ME) extraction. The FE was conducted following the guidelines reported elsewhere [16]. Briefly, each FE consisted of 1 g of sample mixed with 20 mL of chloroform:methanol solution (2:1, *v*/*v*). Then, the mixture was vortexed for 5 min followed by centrifugation at 4200 × *g* for 5 min. The lower phase (lipids dissolved in chloroform) was transferred to a test tube, where the chloroform was evaporated at 45 °C under nitrogen flow. The ME was conducted according to methodology reported elsewhere [17] with some modifications. One gram of the sample was transferred into a Mojonnier test tube and diluted with 6 mL of deionized water. The diluted sample was mixed 1.5 mL of NH_4_OH, 10 mL of ethyl alcohol, 25 mL of ethyl ester, 25 mL of petroleum ether, and a few drops of phenolphthalein indicator. Then, the mixture was vigorously shaken and centrifuged for 5 min. After centrifugation, the lipid phase was poured into an aluminum pan, where the organic solvents were evaporated by heating the pan at 65 °C. A second extraction was performed using 5 mL of ethyl alcohol, 15 mL of ethyl ether, and 15 mL of petroleum ether. The dried lipids were weighed, and the extraction efficiency was expressed as the percentage of total lipids recovered according to Equation (1):

(1) $$Total\;lipids\;\left(\%\right)=\frac{Weight\;of\;recovered\;lipids}{Weight\;of\;sample}\cdot 100.$$

### 2.4. Switchable Solvent Extraction

**Figure 1:**
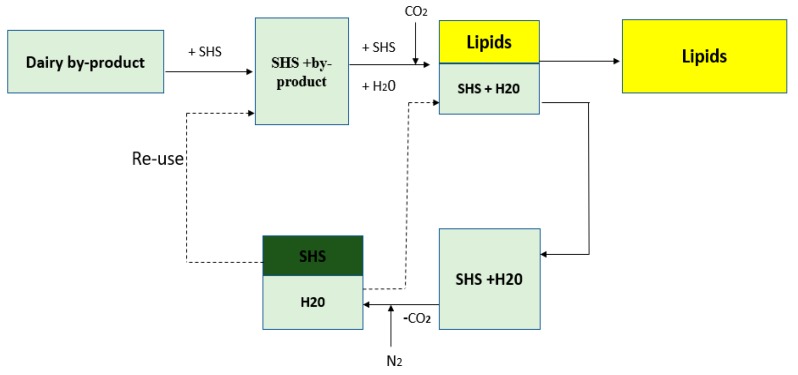
Schematic diagram for the extraction of lipids from dairy byproducts using switchable hydrophilicity solvent (SHS). The dotted lines represent water and SHS that can be reused at the end of the process.

**Figure 2:**
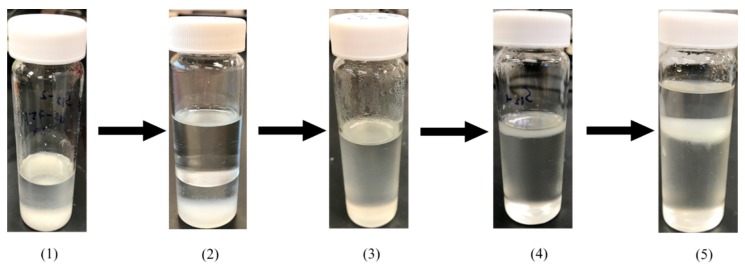
Extraction of lipids from dairy byproducts with N,N-dimethylcyclohexylamine (CyNMe2) as a switchable hydrophilicity solvent (SHS): (**1**) SHS with dairy sample after 18 h of extraction; (**2**) addition of water; (**3**) after bubbling CO_2_ to separate the lipid phase; (**4**) lipids on the top and CyNMe2 on the bottom; and (**5**) addition of hexane to facilitate the separation of lipids.

Figure 1 is a schematic depiction of the lipid extraction via SHS from different dairy matrices. Each extraction consisted of 1 g of sample added into a 20 mL vial containing either 3, 6, or 12 mL of CyNMe2. Figure 2 illustrates the SHS extraction of PLs, where the mixture (solvent/matrix) was stirred at room temperature for 18 h (step (1) in Figure 2). Afterward, an equimolar amount of water was added to maintain the stoichiometry of the reaction (step (2)) followed by bubbling CO_2_ at room temperature (step (3)) until the layer of CyNMe2 and water combined (usually 3–4 h), leaving the lipid layer at the top of the vial. The presence of CO_2_ converted the CyNMe2 into its respective water-soluble salt, switching the hydrophobic form of the amine into the hydrophilic form of the bicarbonate salt where the layer of lipids is at the top of the mixture (step (4)). Three milliliters of hexane was added to dissolve the lipid layer, and subsequently transferred to a test tube. The hydrophilic mixture made of CyNMe2 and water was separated into their respective components by removing the CO_2_ until the layers were visibly formed. The separated hydrophobic CyNMe2 and water were recovered for further lipid extraction.

### 2.5. Fractionation of Extracted Lipids 

The extracted lipids were fractionated via solid-phase extraction (SPE) following the methodology reported elsewhere [18]. An SPE column (1 cm × 10 cm) made of activated silica gel was first conditioned with 10 mL of chloroform:methanol mixture (95:5, *v*/*v*). A portion of the extracted lipids (100 mg) was dissolved in 1 mL of the chloroform:methanol solution and run through the conditioned column. The neutral lipids were eluted with 20 mL of chloroform:methanol (95:5, *v/v*). The PLs were recovered using 10 mL of methanol followed by 10 mL of chloroform:methanol:water mixture (5:3:2, *v*/*v*/*v*). Afterward, the solvents from the PLs fraction were evaporated under vacuum at 40 °C. The recovered PLs were expressed as a percentage of the extracted lipids, according to Equation (2):

(2) $$Recovered\;phospholipids\;\left(\%\right)=\frac{Weight\;of\;dried\;fraction}{Weight\;of\;lipids}\cdot 100.$$

### 2.6. Thin-Layer Chromatography (TLC) 

The recovered PLs were quantified on TLC silica gel plates. A mixture of chloroform:methanol:water (65:25:4, *v*/*v*) was used as a mobile phase for the detection of PLs. A solution of molybdophosphoric acid (5%, *w*/*v*) in ethanol was used as spray reagent, followed by heating at 180 °C for 10 min [19]. Clear visualization of the detection of spots was obtained under UV light.

### 2.7. Statistical Analysis

Each extraction was carried out in triplicate, and the mean values for total lipids and recovered PLs were compared using Tukey’s test (*p* < 0.05). The statistical analysis was carried out using Sigmaplot software V11 (SPSS Inc., Chicago, IL, USA).

## 3. Results

### 3.1. Compositional Analysis

Table 1 shows the compositional characteristics of the different dairy matrices used in this study. The evaluated parameters were within the expected ranges for each matrix. On the other hand, substantial variations in the total solids (8%–91%), lactose (3%–8%), and fat (4%–27%) were observed between the different matrices. Such variations allowed us to evaluate the feasibility of SHS in extracting PLs under different environmental factors.

### 3.2. Total Lipid Extraction

**Figure 3:**
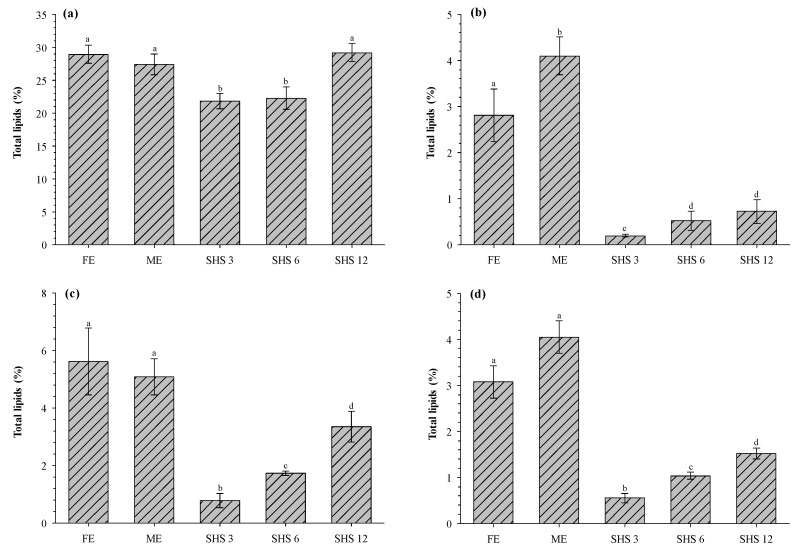
Comparison of different extraction methods (Folch, Mojonnier, and switchable hydrophilicity solvent) on the lipids recovered from (**a**) raw cream, (**b**) buttermilk, (**c**) concentrated buttermilk, and (**d**) beta-serum. FE: Folch extraction; ME: Mojonnier extraction; SHS 3, 6, 12: switchable hydrophilicity solvent ratio 1/3, 1/6, 1/12, respectively. Mean ± standard deviation within each column with different letters (**a**–**d**) are significantly different (*p* < 0.05) according to Tukey test.

The extracted total lipids from different dairy matrices (RC, BM, CBM, and BS) using three different methods (FE, ME, and SHS) are shown in Figure 3. Overall, the type of dairy matrix strongly influenced the amount of total lipids extracted. For RC (Figure 3a), the FE and ME showed no significant difference in the amount of total lipids recovered (28.98% ± 1.36% and 27.47% ± 1.60%, respectively). In contrast, lower values of total lipids were obtained with CyNMe2 at SHS/RC ratios 3/1 and 6/1 (21.84% ± 1.15% and 22.32% ± 1.75%, respectively). On the other hand, the total lipids recovered from RC increased to 29.24% ± 1.38% with increasing the SHS/RC ratio to 12/1. Such values of extracted total lipids were not significantly different from those obtained with FE and ME (Figure 3a). Moreover, FE and ME required a substantially higher ratio of the solvent/matrix (20/1 and 50/1, respectively) than that of the SHS/RC of 12/1. Further increment of the SHS/sample ratio did not result in higher values of extracted total lipids. 

Figure 3b shows the extracted total lipids from BM using FE, ME, and SHS (at 3/1, 6/1, and 12/1 ratios). The highest values of extracted total lipids were obtained using ME (4.09% ± 0.41%), followed by FE (2.81% ± 0.57%). The extraction values via SHS were rather low (0.18%–0.71%), showing an increasing tendency with the solvent/matrix ratio. Similar trends were observed for the extraction of total lipids from CBM (Figure 3c), where the extraction with SHS increased (0.77% ± 0.25% to 3.34% ± 0.54%) with the solvent/matrix ratio. However, the highest extraction was obtained using FE (5.62% ± 1.16%), followed by ME (5.09% ± 0.63%). CBM through either freeze-drying or spray drying has been used as a feedstock for the concentration of lipids using supercritical CO_2_ [5,8,9,10]. Figure 3d shows the extraction of total lipids from BS using FE, ME, and SHS. The highest extraction of total lipids was obtained with ME (4.05% ± 0.35%) followed by FE (3.07% ± 0.35%), while lower values of extraction were obtained with SHS (0.55%–1.52%). 

### 3.3. Phospholipids Recovered

**Figure 4:**
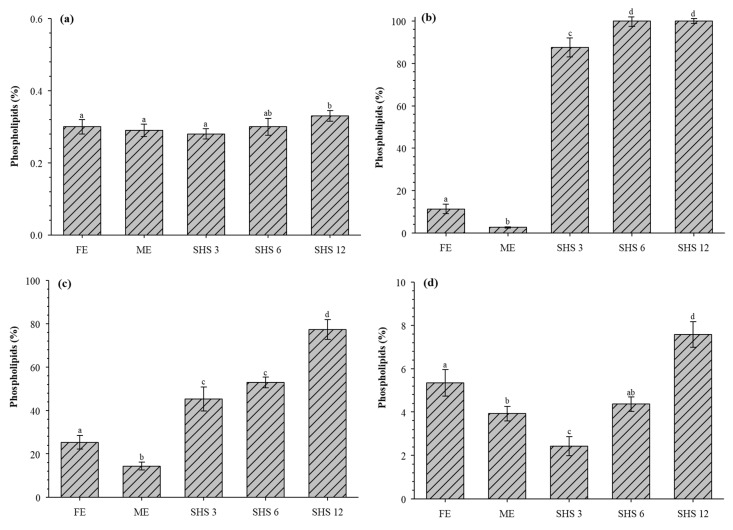
Comparison of different extraction methods (Folch, Mojonnier, and switchable hydrophilicity solvent) on the phospholipids recovered from (**a**) raw cream, (**b**) buttermilk, (**c**) concentrated buttermilk, and (**d**) beta-serum. FE: Folch extraction; ME: Mojonnier extraction; SHS 3, 6, 12: switchable hydrophilicity solvent ratio 1/3, 1/6, 1/12, respectively. Mean ± standard deviation within each column with different letters (**a**–**d**) are significantly different (*p* < 0.05) according to Tukey test.

The extracted total lipids were fractionated with SPE to calculate the amount of recovered PLs. Figure 4 shows the recovered PLs from different dairy matrices (RC, BM, CBM, and BS) using three different methods (FE, ME, and SHS). In the case of RC, the recovered PLs with FE, ME, and SHS 3/1 ranged from 0.28%–0.30%, and no significant difference was detected between extraction methods. On the other hand, the recovered PLs with SHS 12/1 were slightly higher (0.33% ± 0.01%) than any other extraction method. Other authors have reported similar values of recovered PLs (0.40%) from cream using FE [20]. 

The recovered PLs from BM (Figure 4b) using SHS were remarkably higher compared with those obtained from FE and ME. The use of CyNMe2 as a SHS remarkably extracted 87.50% ± 4.50%, 99.93% ± 2.50%, and 99.96% ± 1.21% at ratios 3/1, 6/1, and 12/1, respectively (Figure 4b). In contrast, only 11.37% ± 2.31% and 2.66% ± 0.26% of the PLs were recovered using FE and ME, respectively. In the case of CBM, 25.33% ± 3.10% and 14.41% ± 1.78% of the PLs were recovered using FE and ME, respectively. Interestingly, the use of SHS substantially increased the amount of recovered PLs (45.21% ± 5.51%, 52.84% ± 2.45%, and 77.27% ± 4.51% at ratios of 3/1, 6/1, and 12/1, respectively. Figure 4d shows the recovered PLs from BS, where the highest recovered amount was obtained using SHS 12/1 (7.57% ± 0.59%) followed by FE (5.34% ± 0.61%). In contrast, the lowest amount of recovered PLs was found in the samples extracted with SHS 3/1 (2.41% ± 0.44%) and ME (3.92% ± 0.33%).

### 3.4. PLs TLC Characterization 

**Figure 5:**
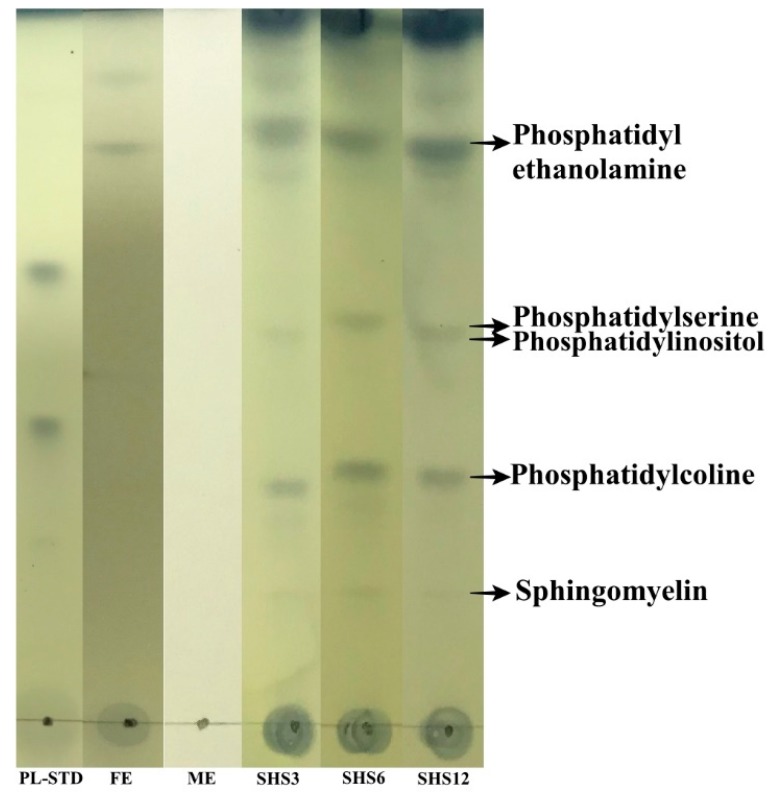
Representative thin-layer chromatography (TLC) plate of buttermilk showing the migration of the recovered phospholipids. PL-STD: the standard mixture of phospholipids; FE: Folch extraction; ME: Mojonnier extraction; SHS 3, 6, 12: switchable hydrophilicity solvent ratios 1/3, 1/6, 1/12, respectively.

A representative TLC image of the buttermilk PLs is shown in Figure 5. There are four visible color bands in the TLC plate, which represent the individual PLs (phosphatidyl ethanolamine, PE; phosphatidylserine, PS; phosphatidylinositol, PI; phosphatidylcholine, PC; and sphingomyelin, SM). Similar results also confirmed the presence of individual PLs for raw cream, buttermilk powder, and processed milk [17]. The recovered PLs using SHS clearly outperformed the conventional extraction methods, judging by the bands corresponding to individual PLs.

## 4. Discussion

The extraction with CyNMe2 as an SHS resulted in a higher amount of recovered PLs, regardless of the dairy matrix, with recovery values ranging from 0.33% to 99%. Traditional extraction methods such as FE combine organic solvents with polar alcohol that disrupts the hydrogen bonding and electrostatic forces between the polar lipids and proteins, creating holes in the membrane. Such a combination of organic solvent and polar alcohol somehow enables the non-polar solvent to enter the cells and interact with the hydrophobic neutral lipids [21]. 

**Figure 6:**
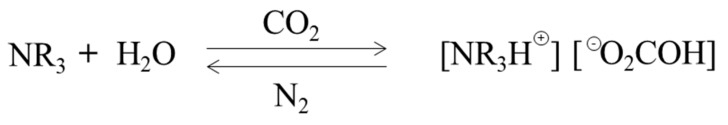
Reaction mechanism from switching hydrophilicity of N,N-dimethylcyclohexylamine. Adapted from [21].

In the ME, a strong base (NH_4_OH) is used to digest the protein and release the fat, which is subsequently dissolved with a mixture of organic solvents [17]. However, the majority of PLs remained within the ammonia phase rather than the organic solvent phase. Remarkably, the extraction using CyNMe2 as an SHS resulted in higher values of recovered PLs despite not having polar alcohol or a denaturing agent. CyNMe2 is a commercially available tertiary amine that has very low miscibility with water under nitrogen atmosphere (atmospheric conditions), and it becomes hydrophilic in the presence of CO_2_. In the presence of CO_2_, CyNMe2 forms a water-soluble bicarbonate salt, while in the absence of CO_2_ the carbonated salt is converted back into CyNMe2 [20]. The chemical reaction responsible for the immiscibility change is represented in Figure 6. 

The efficiency of CyNMe2 in extracting PLs directly from dairy matrices was tested by varying the solvent/matrix ratio. The majority of research using a tertiary amine as an SHS involves the use of CyNMe2. This has been exemplified elsewhere [14], and CyNMe2 was used to extract and isolate lipids including triacylglycerol, diacylglycerol, monoacylglycerol, and free fatty acids from algae (*Botryococcus braunii*). These authors reported values of recovered lipids up to 22% of the dry cell weight at room temperature. The use of CyNMe2 for extracting PLs from raw cream was rather low (0.28%–0.31%). This is because the PLs along with the membrane proteins emulsify the fat, creating thick cell walls that make the extraction of PLs very difficult. The extraction of PLs from RC may be improved by the application of mechanical treatment prior to extraction, which is beyond the scope of this work. 

The performance of the SHS in directly extracting PLs from BM was quite remarkable, judging by the unprecedented amount of recovered PLs (99%). Direct extraction of PLs from a dilute stream offers logistical and financial advantages over conventional organic solvent extraction methods. The extraction of lipids directly from dilute media without pretreatment has been documented elsewhere [22], in which CyNMe2 was used to extract lipids directly from wet algae (up 80% water content), and extraction yields in the range of 31%–57% were reported. 

The current literature on the utilization of dairy PLs deals with the development of concentrates as end-product, and no information is available regarding isolated fractions of PLs. In the absence of such information, reports dealing with the concentrates of dairy PLs obtained through a combination of technologies (enzymatic hydrolysis, microfiltration, ultrafiltration, and supercritical carbon dioxide) were used for comparison with our experimental findings. A two-step process for obtaining a concentrate of PLs (up to 19%) from reconstituted buttermilk using microfiltration and supercritical carbon dioxide extraction has been developed [8]. Similarly, other authors concentrated 56% of PLs from reconstituted buttermilk using enzymatic hydrolysis of protein prior to microfiltration followed by supercritical carbon dioxide with ethanol as co-solvent [9]. A concentrate containing 60% of PLs was obtained by ultrafiltration of whey buttermilk prior to spray drying. Then, the final concentration of PLs was obtained after extraction using supercritical carbon dioxide [5]. An investigation on the concentration of PLs from buttermilk powder showed a five-fold increment in the PL content by microfiltration coupled with supercritical carbon dioxide [10]. More recently, Price et al. [11] extracted 58% of the total PLs from whey protein phospholipid concentrate using simultaneous texturization and extraction (STEP) under optimum conditions (five-stage sequential extraction using ethanol at 70 °C). All these approaches have led to the enrichment of PLs in the byproduct stream rather than extraction from their respective matrix. We investigated an alternative way to concentrate and isolate dairy PLs, where the values of recovered PLs were 0.33% ± 0.01%, 7.57% ± 0.59%, 77.27% ± 4.51%, and 99.96% ± 1.20% for RC, BS, CBM, and BM, respectively. Our results are in agreement with previous reports elsewhere [22], suggesting that SHS can outperform conventional extraction and concentration methods by directly extracting lipids from dilute streams. Further quantification of the individual PLs would be of relevant interest in future investigations. Research in this area is scarce, and it offers opportunities for further studies in the field of process development, micromixing, optimization, and the development of new types of SHS. It is worth mentioning that CyNMe2 (N,N-dimethylcyclohexylamine) is an amine authorized by the FDA as an Indirect Food Additive (CFR:177.2600). Nevertheless, other relevant aspects need further investigation, such as amine recovery and purity of the PLs fraction, as well as structural modification to minimize potential impact in biological systems. The application of other amines as SHSs is an active research area, including dimethylethanolamine, spermine, and spermidine. 

## 5. Conclusions

For the first time, the feasibility of extracting phospholipids directly from dairy byproducts was evaluated using CyNMe2 as an SHS. The SHS extracted up to 99.96% of the PLs directly from BM, while only between 2% and 11% of the PLs were extracted with conventional methods. Further optimization of other extraction parameters (temperature, time, type of system, and matrix) is needed for the development of solvent systems that maximize the quality of the separations. 

## Figures and Tables

**Table 1:** Composition of dairy byproducts used for the extraction of phospholipids.

Parameter	Dairy Matrix			
	Raw Cream	Butter Milk	Concentrated Butter Milk	B-Serum
Total Solids (%)	51.77 ± 0.18	9.63 ± 0.03	91.81 ± 0.06	8.17 ± 0.34
Protein (%)	0.24 ± 0.01	0.33 ± 0.01	1.44 ± 0.07	0.18 ± 0.01
Fat (%)	27.47 ± 1.60	4.09 ± 0.41	5.09 ± 0.63	4.05 ± 0.04
Ash (%)	0.61 ± 0.02	0.94 ± 0.08	6.26 ± 0.17	1.04 ± 0.39
Lactose (%)	3.06 ± 2.73	3.98 ± 0.39	8.69 ± 0.28	6.91 ± 0.15
pH	6.58 ± 0.01	6.38 ± 0.01	5.05 ± 0.02	6.74 ± 0.01
